# Effectiveness and safety of nurse-led early cognitive and sensory rehabilitation in patients with severe traumatic brain injury: a systematic review protocol

**DOI:** 10.3389/fneur.2025.1659712

**Published:** 2025-10-02

**Authors:** Yandi Wen, Qiaoxia He, Lan Qin, Yuluo Du, Hongyan Yin, Xiaojuan Xiang, Yisong Xie

**Affiliations:** Shenzhen People’s Hospital, Shenzhen, China

**Keywords:** severe traumatic brain injury, cognitive rehabilitation, sensory stimulation, nurse-led, early intervention

## Abstract

**Background:**

Recovery from severe traumatic brain injury (sTBI) is frequently compromised by profound and prolonged disorders of consciousness. While critical care nurses are uniquely positioned to deliver early, structured cognitive and sensory rehabilitation at the bedside, the effectiveness and safety of such nurse-led protocols remain uncertain due to a lack of synthesized evidence. This critical knowledge gap hinders the standardization of neuro-rehabilitative nursing and the optimization of patient recovery trajectories.

**Objectives:**

The primary objective is to systematically evaluate the effectiveness of nurse-led early cognitive and sensory interventions on consciousness recovery and cognitive function in adult sTBI patients. The co-primary objective is to assess the safety of these interventions. Secondary objectives include impacts on functional status, length of ICU/hospital stay, and mortality.

**Methods:**

This systematic review will adhere to the PRISMA-P guidelines and is registered with PROSPERO (CRD420251075729). We will systematically search major international and Chinese databases for relevant randomized controlled trials (RCTs) and non-randomized studies of interventions (NRSI) involving adult patients with severe traumatic brain injury (sTBI), defined as an initial post-resuscitation Glasgow Coma Scale (GCS) score of 8 or less. Two reviewers will independently screen studies, extract data, and assess risk of bias using the Cochrane RoB 2 and ROBINS-I tools. Data will be synthesized narratively. Where appropriate, random-effects meta-analyses will be performed. Pre-specified subgroup and sensitivity analyses will be conducted to explore heterogeneity and assess the robustness of the findings. The final certainty of evidence will be assessed using the GRADE framework.

**Conclusion:**

This review aims to deliver a definitive synthesis of evidence to directly inform the development and implementation of nurse-led neuro-rehabilitation protocols. By establishing the balance of effectiveness and safety, our findings will provide a rigorous foundation to empower nursing practice, enhance neurocritical care, and ultimately, improve the outcomes for this vulnerable patient population.

## 1 Introduction

Traumatic brain injury (TBI) is a major global health concern, imposing a profound burden with millions affected annually (1). Severe TBI (sTBI) frequently leads to devastating long-term neurological impairments, particularly cognitive and sensory deficits (2). Early intervention during the acute phase is critical for mitigating these effects and optimizing functional recovery (3). However, many sTBI patients, especially those with prolonged disorders of consciousness, receive suboptimal early rehabilitation due to various barriers, despite the recognized importance of brainstem function and arousal (4, 5).

Nurses are uniquely positioned to deliver early rehabilitation interventions due to their continuous bedside presence and holistic care approach, encompassing physical, psychological, and social dimensions (6). This constant interaction enables timely assessment, individualized stimulation, and consistent reinforcement, all crucial for neurological recovery in this vulnerable population (7, 8). Consequently, nurse-led early cognitive and sensory rehabilitation, sometimes termed “coma stimulation,” has emerged as a promising strategy to improve outcomes in sTBI patients (9).

Despite this compelling rationale, the clinical evidence for nurse-led protocols remains fragmented, with their specific effectiveness and safety not well-established (10). This evidence gap contributes to significant variations in care, leaving clinicians without a definitive, synthesized foundation to guide practice. Uncertainty persists due to a lack of standardized protocols, concerns over precipitating physiological instability (e.g., increased intracranial pressure) (11), and the practical challenges of integrating these duties into an already demanding nursing workload (12). Given these challenges, clinicians remain uncertain about the optimal components, timing, and intensity of these interventions. A rigorous systematic review is therefore urgently required to systematically consolidate and critically appraise the existing literature, providing the robust evidence needed to inform clinical decision-making and standardize care (13).

## 2 Methods

### 2.1 Study registration

This systematic review protocol has been registered prospectively with the International Prospective Register of Systematic Reviews (PROSPERO), registration number: CRD420251075729. Any subsequent amendments to this protocol will be tracked and explicitly documented in the PROSPERO registration record.

### 2.2 Eligibility criteria

Studies were selected for inclusion based on the following pre-specified criteria for study design, intervention, and comparators.

#### 2.2.1 Types of studies

This review will include randomized controlled trials (RCTs). In recognition of the potential scarcity of RCTs in this critically ill population, and to ensure a comprehensive synthesis of the evidence, we will also include non-randomized studies of interventions (NRSI). Eligible NRSI designs include quasi-randomized trials, controlled before-and-after studies, and non-randomized controlled trials with a concurrent control group (14).

#### 2.2.2 Types of interventions

The eligible intervention is nurse-led early cognitive and sensory rehabilitation, defined as a structured protocol that is:

Nurse-led: This implies that nurses hold primary responsibility for the assessment, initiation, continuous delivery, titration of the therapeutic dose, and ongoing monitoring of the intervention. While other team members (e.g., physiotherapists, occupational therapists) may collaborate or provide consultative input, the direct implementation and day-to-day management of the specified cognitive and sensory rehabilitation protocol must primarily rest with nursing staff.
Early: Initiated during the acute care phase within a critical care or high-dependency unit. We will operationally define “early” as initiated within 14 days post-injury, while also including studies that define their intervention as “early” and exploring timing as a factor in subgroup analysis.
Therapeutic: Explicitly designed to improve neurological outcomes, such as consciousness or cognition.
Components of cognitive and sensory rehabilitation: Interventions must include structured cognitive tasks (e.g., simple command following, orientation to person/place/time, memory recall exercises) and/or multimodal sensory stimulation (e.g., presenting familiar sounds/voices, tactile stimulation with varied textures, visual tracking, olfactory stimulation with meaningful scents). We will seek studies that clearly describe the types of tasks, sensory modalities used, frequency, duration, and intensity of stimulation. The review will include studies using established protocols or standardized tools for delivering these interventions.

#### 2.2.3 Types of comparators

The comparator group will receive standard neuro-ICU care as defined in the primary studies. To systematically manage the inherent variability in this definition, we will categorize the “standard care” comparator during data extraction based on its explicitly reported components. For example, we will distinguish between: (a) baseline neuro-monitoring and life support only; (b) baseline care plus non-protocolized, unstructured family or staff interaction; and (c) baseline care plus other routine rehabilitation (e.g., physical therapy) not directed by the study protocol. This categorization will be used as a basis for subgroup analysis to investigate its impact as a potential source of heterogeneity.

#### 2.2.4 Type of outcome measure

To be eligible for inclusion, studies must report on at least one of the following pre-specified primary or secondary outcomes.

##### 2.2.4.1 Primary outcomes

Our primary outcomes distinctly address both effectiveness and safety:

Effectiveness endpoint: Measures of consciousness and cognition, assessed using any validated instrument, such as the GCS, Coma Recovery Scale-Revised (CRS-R), Rancho Los Amigos Levels of Cognitive Function (RLALCF), Mini-Mental State Examination (MMSE), or direct clinical measures like time to follow commands or time to orientation (15).
Safety endpoint: Safety and adverse events. Safety will be assessed in two ways: (1) We will extract any adverse event explicitly attributed to the intervention by the study authors. (2) To ensure a more systematic assessment, we will proactively screen for and extract data on a pre-specified list of potential adverse events plausibly related to stimulation, including but not limited to: sustained intracranial pressure (ICP) elevation (e.g., >20 mmHg for >5 min), clinically significant drops in cerebral perfusion pressure (CPP), new-onset seizures, paroxysmal sympathetic hyperactivity (PSH), or agitation requiring an increase in sedation.

##### 2.2.4.2 Secondary outcomes

These include:

Global functional status: Measured by validated scales like the Glasgow Outcome Scale-Extended (GOSE), Functional Independence Measure (FIM), or Modified Rankin Scale (mRS) (16).
Health service metrics: Such as the length of stay in the ICU or hospital and the total duration of mechanical ventilation.
Mortality: All-cause mortality at any reported time point (e.g., in-hospital, 30-day, 6-month).
ICU-related complications: Incidence of conditions such as delirium, pneumonia, or ventilator-associated pneumonia.

##### 2.2.4.3 Time points

To distinguish between short-term efficacy and the durability of effects, outcomes will be analyzed at two distinct, pre-specified time points:

Short-term/in-hospital effect: Defined as the first outcome assessment conducted while the patient is still in the ICU, or within 30 days from injury onset, or within 30 days after the completion of the intervention if the intervention duration is clearly defined and consistent. This time point is intended to capture the primary efficacy of the intervention.
Long-term follow-up effect: Defined as the outcome assessment conducted at 3 months or later (≥3 months) from the date of injury. This time point is intended to evaluate the sustainability of any observed benefits. If a study reports multiple long-term follow-ups, we will prioritize data from the time point closest to 6 months for the primary analysis to enhance consistency across studies. Data from significantly later time points (e.g., >12 months) will be extracted and presented narratively, or if sufficient studies exist, considered for a separate meta-analysis.

#### 2.2.5 Type of exclusion criteria

Studies will be explicitly excluded if they meet one or more of the following criteria:

Population: To ensure a focused analysis on the target population, studies will be excluded if they focus exclusively on pediatric populations (<18 years) or non-TBI diagnoses (e.g., stroke, anoxia) where TBI subgroup data are not reported separately. Our operational definition of severe TBI is an initial post-resuscitation Glasgow Coma Scale (GCS) score of 8 or less. Consequently, studies focusing exclusively on cohorts with mild or moderate TBI will be excluded.
Intervention: Protocols that are not nurse-led (e.g., primarily delivered by physiatrists or occupational therapists), interventions focused solely on physical mobilization without a structured cognitive/sensory component, or unstructured interactions considered part of standard care.
Study design: Case reports, case series, observational studies that lack a comparator group, literature reviews, editorials, and conference abstracts.
Setting: Studies will be excluded if the intervention is initiated primarily in a post-discharge or outpatient setting. Our focus is on early interventions delivered within acute care (ICU or high-dependency unit) or inpatient rehabilitation facilities during the acute or subacute phase of recovery.
Language: Articles for which a high-quality translation from a non-English language cannot be feasibly obtained will be excluded, and this will be documented.

### 2.3 Search methods for identification of studies

#### 2.3.1 Electronic data sources

A comprehensive electronic literature search will be conducted to identify relevant studies by systematically searching the following international and Chinese databases: PubMed, Embase, the Cochrane Central Register of Controlled Trials (CENTRAL), Web of Science, Scopus, China National Knowledge Infrastructure (CNKI), Wanfang Database, VIP Information (VIP), and the China Biomedical Literature Database (CBM). The search will cover the period from January 1, 2000, to the date of search execution (anticipated in July 2025), and no language restrictions will be applied during the search phase. For each database, search strategies will be developed using an appropriate combination of controlled vocabulary (e.g., MeSH, Emtree) and relevant keywords related to severe traumatic brain injury, nurse-led interventions, and early cognitive or sensory rehabilitation.

#### 2.3.2 Searching other resources

To identify all relevant research and mitigate publication bias, we will supplement our database search by manually scanning the reference lists of included studies and relevant systematic reviews. Furthermore, we will search major clinical trial registries (e.g., ClinicalTrials.gov, ISRCTN) for completed but unpublished studies, and search ProQuest Dissertations & Theses Global for relevant doctoral dissertations.

#### 2.3.3 Search strategy

See Supplementary material for details.

### 2.4 Data collection and analysis

#### 2.4.1 Selection of studies

**Figure 1:**
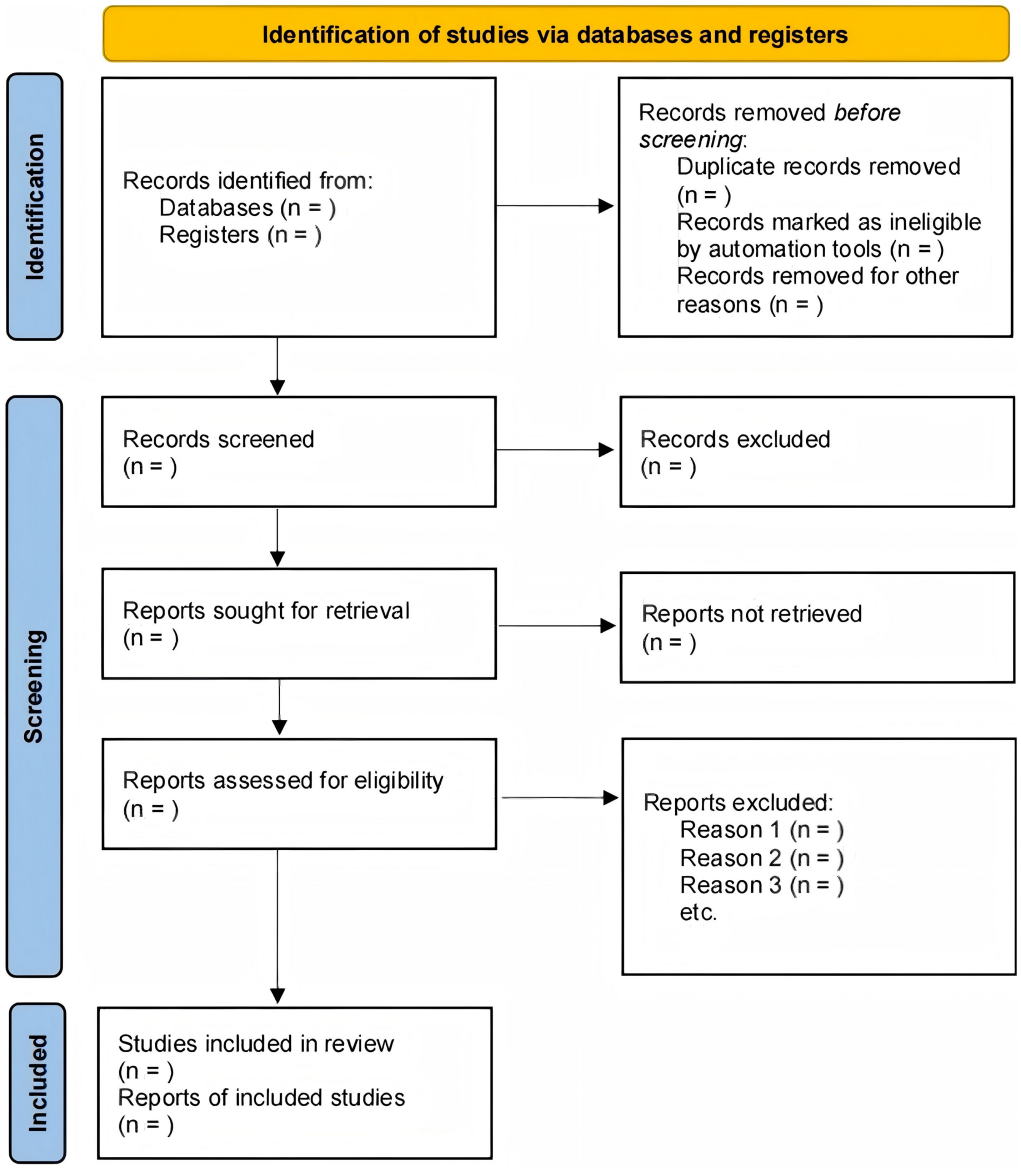
Flowchart of the literature screening process.

The study selection will be conducted and reported in accordance with the Preferred Reporting Items for Systematic Reviews and Meta-Analyses (PRISMA) statement. After search results are aggregated, duplicates will be removed using reference management software (e.g., EndNote), and the remaining records will be imported into a systematic review platform (e.g., Covidence) to manage the screening process (Figure 1).

Two reviewers will independently execute a two-phase screening process. First, they will screen the titles and abstracts of all identified records; any citation deemed potentially relevant by at least one reviewer will advance. Second, the same two reviewers will independently assess the full text of these records against the pre-specified eligibility criteria for final inclusion. Any discrepancies at either screening stage will be resolved by discussion to reach a consensus. If an agreement cannot be reached, a third, senior reviewer will be consulted for final adjudication. The entire selection process will be documented in a PRISMA flow diagram, which will detail the number of studies at each stage and provide reasons for the exclusion of studies at the full-text review level.

#### 2.4.2 Data extraction and management

To ensure systematic and consistent data collection, a standardized data extraction form will be developed in a spreadsheet program (e.g., Microsoft Excel). The form will be piloted by two reviewers on a sample of three included articles and refined to ensure clarity and comprehensiveness. The entire data extraction process will then be conducted independently and in duplicate by two reviewers to minimize error and bias. Any discrepancies will be resolved through discussion to reach a consensus; if an agreement cannot be reached, a third, senior reviewer will provide final adjudication.

The extraction form will be structured to capture comprehensive information across key domains, including: study characteristics (e.g., author, year, funding sources); population details (e.g., sample size, inclusion criteria, baseline demographics); detailed intervention specifics (e.g., components, dosage, timing, nurse training); a clear description of the comparator “standard care”; and all pre-specified outcome data. For quantitative synthesis, the extraction of outcome data will include the measurement tools, assessment time points, reported effect estimates, and the raw data required for meta-analysis (i.e., the number of events and participants for dichotomous outcomes, and means with standard deviations for continuous outcomes).

In cases where data are unclear, missing, or presented in a format that precludes synthesis (e.g., graphs without raw data), we will contact the corresponding authors by email up to two times to request the necessary information. All extracted data will be managed in a central, password-protected database accessible only to the review team.

#### 2.4.3 Assessment of risk of bias in included studies

The methodological quality of each included study will be rigorously assessed to evaluate the confidence in its findings. This process will be conducted independently by two reviewers. The choice of assessment tool will be tailored to the study design: the Cochrane Risk-of-Bias 2 (RoB 2) tool will be used for RCTs, while the Risk of Bias in Non-Randomized Studies-of Interventions (ROBINS-I) tool will be used for non-randomized studies. For each study, reviewers will assign a judgment of “Low risk of bias,” “Some concerns,” or “High risk of bias” for each domain and for the overall study. Any disagreements between reviewers will be resolved through discussion to reach a consensus; a third, senior reviewer will serve as an arbiter if necessary.

The results of this assessment will be presented visually in a summary table and a “traffic-light” plot to provide a clear overview of the quality across all included studies. The risk of bias assessment will not be used as a criterion for excluding studies. Instead, it will be integral to the interpretation and synthesis of the evidence in three key ways: (1) it will be considered in the narrative synthesis of the findings; (2) it will be used to conduct sensitivity analyses (e.g., re-running meta-analyses excluding studies at high risk of bias); and (3) it will directly inform the “risk of bias” domain when grading the overall certainty of the body of evidence for each outcome using the GRADE framework.

#### 2.4.4 Data synthesis

We will first conduct a narrative synthesis of the data extracted from the included studies to systematically summarize their basic characteristics and key findings. Subsequently, if the studies are found to be sufficiently comparable and homogeneous in terms of study design, interventions, populations, and outcomes, a quantitative synthesis, i.e., a meta-analysis, will be performed. For dichotomous outcomes, we will calculate the odds ratio (OR) or risk ratio (RR), while for continuous outcomes, the mean difference (MD) or standardized mean difference (SMD) will be calculated, both reported with their 95% confidence intervals (95% CI). A random-effects model will be preferentially used for data pooling to account for potential inherent differences across studies. Statistical heterogeneity will be assessed using the Cochran’s *Q* test and the *I*^2^ statistic, where a *p*-value <0.10 for the *Q* test or an *I*^2^ > 50% will be considered to indicate significant heterogeneity. If significant heterogeneity is detected (*I*^2^ > 50%), we will explore its potential sources through the pre-specified subgroup analyses. If a sufficient number of studies are available (typically ≥10 studies per covariate), we will also consider conducting an exploratory random-effects meta-regression to investigate the influence of the following continuous study-level variables: (a) mean baseline GCS score; (b) cumulative weekly intervention dose (in hours); and (c) mean patient age. To examine the robustness of the pooled results, we will conduct a sensitivity analysis, for instance, by sequentially removing individual studies (leave-one-out method) or altering the statistical model (e.g., comparing the random-effects with a fixed-effect model). Potential publication bias will be assessed by visual inspection of a funnel plot, supplemented by Egger’s or Begg’s test (17). All data analyses will be performed using Stata 17.0 or Review Manager 5.4.

#### 2.4.5 Dealing with missing data

We will employ a systematic approach to manage missing data and minimize potential bias. For any included study with missing information, our primary strategy will be to contact the corresponding authors by email up to two times to request the necessary outcome data or summary statistics. Our analytical approach for any remaining missing data will be as follows: attrition (missing participant data) will be evaluated as part of the risk of bias assessment, and our primary analysis will be based on available data, prioritizing intention-to-treat (ITT) results where reported. If a study has high attrition (e.g., >20%) for a primary outcome, we will conduct a best-case/worst-case sensitivity analysis to assess its impact. For missing statistical data (e.g., standard deviations), we will use established methods, as recommended by the Cochrane Handbook, to estimate them from reported confidence intervals, *p*-values, or medians and interquartile ranges. All assumptions, calculations, and imputations will be transparently reported, and their potential influence on the review’s conclusions will be critically discussed.

#### 2.4.6 Subgroup analysis

To investigate anticipated sources of heterogeneity and answer key clinical questions, we will conduct the following mandatory, pre-specified subgroup analyses, provided sufficient data (at least two studies per subgroup) are available:

Intervention components: We will stratify studies based on the primary focus of the intervention: (a) primarily sensory stimulation (e.g., auditory, tactile) versus (b) combined cognitive and sensory protocols. Justification: This analysis seeks to determine if there is a differential effect between targeting basic arousal pathways versus more complex cognitive functions.
Intervention “Dose”: Studies will be grouped by the total daily duration of the intervention: (a) high-intensity (e.g., >60 min/day) versus (b) low-intensity (≤60 min/day). Justification: This analysis aims to explore a potential dose–response relationship to inform clinical practice recommendations.
Timing of intervention initiation: We will analyze subgroups based on when the intervention began: (a) very early acute phase (initiated ≤72 h post-injury); (b) early acute phase (initiated >72 h to 7 days post-injury); versus (c) late acute phase (initiated >7 days to 14 days post-injury). Justification: This more granular stratification allows for a nuanced exploration of the potential confounding influence of very early spontaneous recovery and differing neuroplasticity windows on treatment effects.
Overall risk of bias: Studies will be stratified as “Low risk of bias” versus “Some concerns/High risk of bias” to assess the impact of study quality.

#### 2.4.7 Sensitivity analysis

To assess the robustness of our findings, we will conduct several pre-specified sensitivity analyses. Primarily, we will conduct a critical sensitivity analysis to address the high risk of attrition bias inherent in the severe TBI population. This will involve repeating the primary meta-analysis including only studies judged to have a “Low risk of bias” in the “Bias due to missing outcome data” domain or those that used robust statistical methods (e.g., multiple imputation) to handle missing data.

Additional standard sensitivity analyses will include:

Excluding all studies judged to be at an overall “High risk of bias” to evaluate the impact of lower-quality evidence.
Changing the statistical model (from a random-effects to a fixed-effect model).
Performing a leave-one-out analysis, where we systematically remove one study at a time.

Our findings will be considered robust if the direction and magnitude of the effect estimate remain stable across these different analytical assumptions.

### 2.5 Patient and public involvement

This systematic review was developed with input from patient and public partners. A patient advisory group, consisting of 10 patients with lived experience of the condition and two caregivers, was involved throughout the research process. The group provided critical feedback during the protocol development stage, particularly in refining the selection of primary and secondary outcomes to ensure their relevance to patients’ daily lives. They reviewed the search strategy to ensure its comprehensiveness and will collaborate in drafting the plain language summary of our findings to facilitate wider dissemination to the community. Their contributions were essential for grounding this review in the real-world needs and priorities of patients.

### 2.6 Dissemination and ethics

#### 2.6.1 Dissemination plan

The findings of this systematic review will be disseminated to a wide range of audiences through multiple channels. Our primary method of dissemination will be the publication of the full review in a leading peer-reviewed academic journal. Furthermore, we plan to present the results at relevant national and international scientific conferences. To ensure the findings are accessible to patients, caregivers, and the general public, a plain language summary will be co-produced with our patient partners and distributed through patient advocacy groups and institutional websites. The evidence generated will also be shared with clinical guideline developers and healthcare policymakers to inform their decision-making processes.

#### 2.6.2 Ethical considerations

This study is a systematic review of previously published literature and, as such, does not require formal ethical approval from a Research Ethics Committee or Institutional Review Board as it does not involve direct contact with human participants or the use of their identifiable personal data. The review will be conducted with rigorous adherence to the principles of scientific integrity. The protocol has been registered with PROSPERO (Registration number: CRD420251075729), and the final manuscript will be reported in accordance with the PRISMA 2020 statement to ensure transparency and completeness. All authors will meet the criteria for authorship as defined by the International Committee of Medical Journal Editors (ICMJE).

## 3 Discussion

### 3.1 Innovation points

This systematic review is designed to offer distinct value and move beyond the existing literature through several critical innovations. Firstly, while other reviews may exist on early rehabilitation, this protocol is, to our knowledge, the first to specifically isolate and evaluate the unique contribution of nurse-led interventions. By focusing explicitly on protocols where nurses are the primary drivers or deliverers of care, we aim to generate a direct, actionable evidence base for advancing nursing practice. This moves beyond simply asking if the intervention works, to understanding the therapeutic role of the nurse, leveraging their 24/7 bedside presence and ability to integrate rehabilitation into the fabric of daily care, thereby informing workforce design and enhancing nursing autonomy in the ICU (18).

Secondly, our protocol is designed to bridge the critical gap between efficacy and effectiveness. By strategically including both RCTs and high-quality NRSI (19), we can construct a more comprehensive and generalizable evidence map. This dual approach allows us to synthesize evidence on the intervention’s efficacy under ideal, controlled conditions (from RCTs) alongside its effectiveness in more typical, heterogeneous, real-world clinical settings (from NRSI), making our findings more directly applicable to frontline clinicians.

Thirdly, we will deconstruct the “black box” of this complex rehabilitation intervention. Instead of producing a single, potentially misleading pooled estimate, our pre-specified, hypothesis-driven subgroup analyses on intervention components (sensory vs. cognitive), dosage (high vs. low intensity), and timing (early vs. late acute phase) are designed to provide granular, nuanced, and clinically meaningful insights (20). This sophisticated analytical approach will help answer the crucial questions of what specific elements of the intervention are most effective, how much is required for a therapeutic effect, and when it should be initiated, offering clinicians far more sophisticated guidance than a simple dichotomous conclusion (21).

Fourthly, our protocol moves beyond treating “standard care” as a monolithic control group. By pre-specifying a framework to categorize the components of the comparator group during data extraction, we can use subgroup analyses to explore how the relative effectiveness of the nurse-led intervention changes depending on the baseline level of care provided. This methodological refinement allows for a more nuanced interpretation of the evidence and provides critical context for implementing findings across diverse clinical settings.

### 3.2 Limitations

We acknowledge several potential limitations inherent to this research question and the existing body of literature, which will be critically considered when interpreting our findings. A primary limitation is the anticipated inclusion of NRSI alongside RCTs. While this approach enhances the review’s comprehensiveness, NRSI carry a higher inherent risk of bias, particularly from confounding by indication and selection bias, which may impact the validity of the pooled effect estimates (22). Even within RCTs, we anticipate significant methodological challenges; the nature of the intervention makes blinding of personnel and patients largely infeasible, creating a high risk of performance and detection bias. Furthermore, the severe TBI population is notoriously prone to high rates of attrition, which can lead to significant bias from missing outcome data if not handled appropriately by primary study authors.

Significant clinical and statistical heterogeneity is expected and represents a fundamental challenge. This diversity will likely stem from wide variations in the operational definitions of “nurse-led” interventions, the components and “dosage” (intensity, frequency, duration) of rehabilitation protocols, the evolving nature and variability of the “standard care” comparator across different eras and institutions, and the multitude of outcome measures used (23). This may limit the appropriateness of meta-analysis for some outcomes and necessitate a greater reliance on narrative synthesis.

Lastly, the body of evidence may be susceptible to reporting biases. Beyond publication bias, where entire studies with negative or null findings may remain unpublished, there is a risk of selective outcome reporting, where we may only report on outcomes that showed a statistically significant effect. While our comprehensive search strategy and formal tests (e.g., Egger’s test) are designed to mitigate and detect some of these issues, they cannot eliminate them entirely. Critically, by systematically identifying and appraising these limitations in the existing literature, our review will not only provide a cautious interpretation of the current evidence but also create a clear, evidence-based roadmap for designing the next generation of more robust, methodologically sound clinical trials in this vital area of research.

### 3.3 Implications for future research and clinical practice

Beyond simply synthesizing past research, this review is designed to actively shape the future of neuro-rehabilitative nursing. The anticipated findings will have direct and multifaceted implications. For clinical practice, by delineating the effectiveness, safety, and optimal parameters (e.g., dose, timing) of nurse-led interventions, our review will provide the high-quality evidence needed to develop standardized, institution-wide protocols.

This can empower critical care nurses, moving their role from task-based care towards autonomous, evidence-based therapeutic intervention, and potentially improve consistency of care.

Beyond direct neurological improvements, nurse-led early rehabilitation is anticipated to positively influence patients’ overall well-being, consistent with the concept that improving physical fitness can benefit both cardiovascular and cognitive functions, influencing “hearts and minds” (24). Patients with sTBI frequently experience significant mental and psychological stress; similar to observations of cognitive and mental health disturbances in other vulnerable populations during periods of significant stress (25), sTBI patients are highly susceptible to psychological distress and cognitive deficits. Therefore, early, structured interventions may also contribute to psychological resilience. Indeed, regular physical activity has been associated with a lower risk of cognitive impairment in the general population (26), suggesting that early cognitive and sensory rehabilitation in sTBI patients may similarly contribute to improved cognitive outcomes. Furthermore, given the intricate bidirectional relationship between sleep, sleep disorders, and mental health (27), these interventions might also indirectly promote better sleep quality, further aiding psychological well-being and recovery.

For future research, our systematic appraisal of existing literature, particularly its limitations, will serve as a crucial roadmap. By identifying gaps in evidence and methodological weaknesses in prior studies (e.g., high attrition, inconsistent outcome measures), we will provide specific, evidence-based recommendations for the design of future large-scale, robust randomized controlled trials. This will ensure that the next generation of research is better equipped to provide definitive answers. Ultimately, this systematic review aims not only to fill a knowledge gap but also to catalyze a cycle of evidence-informed practice and practice-informed research, advancing the standard of care and optimizing the recovery for patients with severe TBI.
