# Supplementary material for: Effectiveness and safety of nurse-led early cognitive and sensory rehabilitation in patients with severe traumatic brain injury: a systematic review protocol
Source: Front Neurol. 2025 Oct 2;16:1659712. doi: 10.3389/fneur.2025.1659712 (PMC12527836; doi:10.3389/fneur.2025.1659712)
Supplement: Supplementary file 1 [file Data_Sheet_1.pdf]

Database	Step No	Search Terms
PubMed	1	("Brain Injuries, Traumatic"[Mesh] OR "Craniocerebral Trauma"[Mesh] OR "traumatic brain injury*"[tiab] OR "severe TBI"[tiab] OR "head injury*"[tiab] OR "head trauma"[tiab])
	2	("Cognitive Remediation"[Mesh] OR "Sensation"[Mesh] OR "Communication"[Mesh] OR "Arousal"[Mesh] OR "sensory stimulation"[tiab] OR "multisensory stimulation"[tiab] OR "cognitive stimulation"[tiab] OR "cognitive rehabilitation"[tiab] OR "coma arousal"[tiab] OR "reality orientation"[tiab])
	3	("Nurses"[Mesh] OR "Nursing Care"[Mesh] OR "nurse"[tiab] OR "nursing"[tiab] OR "nurse-led"[tiab])
	4	(Randomized Controlled Trial[pt] OR Controlled Clinical Trial[pt] OR randomized[tiab] OR placebo[tiab] OR trial[tiab] OR groups[tiab]) NOT (animals[mh] NOT humans[mh])
	5	#1 AND #2 AND #3 AND #4
Embase	1	('brain injury'/exp OR 'head injury'/exp OR 'traumatic brain injury*':ti,ab,kw OR 'severe TBI':ti,ab,kw OR 'head trauma':ti,ab,kw)
	2	('cognitive rehabilitation'/exp OR 'sensation'/exp OR 'arousal'/exp OR 'sensory stimulation':ti,ab,kw OR 'multisensory stimulation':ti,ab,kw OR 'cognitive stimulation':ti,ab,kw OR 'coma arousal':ti,ab,kw OR 'reality orientation':ti,ab,kw)
	3	('nurse'/exp OR 'nursing care'/exp OR 'nurse':ti,ab,kw OR 'nursing':ti,ab,kw OR 'nurse led':ti,ab,kw)
	4	('randomized controlled trial'/exp OR 'controlled clinical trial'/exp OR random*:ti,ab,kw OR trial:ti,ab,kw OR placebo:ti,ab,kw)
	5	('animal'/exp NOT 'human'/exp)
	6	#1 AND #2 AND #3 AND #4 NOT #5
Cochrane CENTRAL	1	[mh "Brain Injuries, Traumatic"]
	2	("traumatic brain injury*":ti,ab,kw OR "severe TBI":ti,ab,kw OR "head injury*":ti,ab,kw)
	3	[mh "Cognitive Remediation"] OR [mh Sensation] OR [mh Arousal]
	4	("sensory stimulation":ti,ab,kw OR "multisensory stimulation":ti,ab,kw OR "cognitive stimulation":ti,ab,kw OR "coma arousal":ti,ab,kw)

	5	[mh Nurses] OR [mh "Nursing Care"]
	6	(nurse:ti,ab,kw OR nursing:ti,ab,kw OR "nurse-led":ti,ab,kw)
	7	(#1 OR #2) AND (#3 OR #4) AND (#5 OR #6)
Web of Science	1	TS=("traumatic brain injury*" OR "severe TBI" OR "head injury*") AND TS=("sensory stimulation" OR "multisensory stimulation" OR "cognitive stimulation" OR "cognitive rehabilitation" OR "coma arousal") AND TS=(nurse OR nursing OR "nurse-led") AND TS=(randomized OR randomised OR trial OR controlled OR placebo)
Scopus	1	TITLE-ABS-KEY("traumatic brain injury*" OR "severe TBI" OR "head injury*") AND TITLE-ABS-KEY("sensory stimulation" OR "multisensory stimulation" OR "cognitive stimulation" OR "cognitive rehabilitation" OR "coma arousal") AND TITLE-ABS-KEY(nurse OR nursing OR "nurse-led") AND (LIMIT-TO(DOCTYPE,"ar") OR LIMIT-TO(DOCTYPE,"re")) AND TITLE-ABS-KEY(random* OR trial OR placebo OR controlled)
CNKI	1	TKA%=('颅脑损伤' + '颅脑外伤' + '头部外伤' + '重型颅脑损伤') AND TKA%=('感觉刺激' + '多感官刺激' + '认知康复' + '认知刺激' + '促醒' + '昏迷促醒' + '现实定向') AND TKA%=('护士' + '护理' + '护士主导') AND TKA%=('随机' + '对照' + '临床试验' + '分组') NOT TKA%=('动物' + '大鼠' + '小鼠' + '综述' + 'Meta 分析')
Wanfang	1	主题:(颅脑损伤 or 颅脑外伤 or 头部外伤) AND 主题:(感觉刺激 or 多感官刺激 or 认知康复 or 促醒 or 昏迷促醒) AND 主题:(护士 or 护理 or 护士主导) AND 主题:(随机 or 对照 or 临床试验) NOT 主题:(动物 or 大鼠 or 小鼠 or 综述)
VIP	1	篇关摘：(颅脑损伤 or 颅脑外伤 or 头部外伤)
	2	篇关摘：(感觉刺激 or 多感官刺激 or 认知康复 or 促醒)
	3	篇关摘：(护士 or 护理 or 护士主导)
	4	篇关摘：(随机 or 对照 or 临床试验)
CBM	1	('颅脑损伤'[不加权:扩展] OR '颅脑外伤'[不加权:扩展]) AND ('感觉刺激'[不加权:扩展] OR '认知康复'[不加权:扩展] OR '促醒'[不加权:扩展]) AND ('护士'[不加权:扩展] OR '护理'[不加权:扩展]) AND ('随机' OR '对照') NOT ('动物'[不加权:扩展] OR '综述'[文献类型] OR 'Meta 分析'[文献类型])
ClinicalTrials.g	1	Condition or disease: Traumatic Brain Injury OR Head Injury

ov	2	Other terms: Sensory Stimulation OR Cognitive Rehabilitation OR Coma Arousal OR Nursing
	3	Study type: Interventional Studies (Clinical Trials)
Chictr	1	(颅脑损伤 OR 颅脑外伤) AND (感觉刺激 OR 认知康复 OR 促醒) AND (护士 OR 护理)
	2	研究类型：干预性研究
OpenGrey	1	("traumatic brain injury" OR "head injury") AND ("sensory stimulation" OR "cognitive rehabilitation") AND (nurse OR nursing) AND (trial OR controlled)
WorldCat	1	Subject:("Traumatic brain injuries") AND Subject:("Cognitive therapy" OR "Sensation") AND kw:(nurse OR nursing) AND kw:(trial OR randomized)
PubMed	1	("Brain Injuries, Traumatic"[Mesh] OR "Craniocerebral Trauma"[Mesh] OR "traumatic brain injury*"[tiab] OR "severe TBI"[tiab] OR "head injury*"[tiab] OR "head trauma"[tiab])
	2	("Cognitive Remediation"[Mesh] OR "Sensation"[Mesh] OR "Communication"[Mesh] OR "Arousal"[Mesh] OR "sensory stimulation"[tiab] OR "multisensory stimulation"[tiab] OR "cognitive stimulation"[tiab] OR "cognitive rehabilitation"[tiab] OR "coma arousal"[tiab] OR "reality orientation"[tiab])
	3	("Nurses"[Mesh] OR "Nursing Care"[Mesh] OR "nurse"[tiab] OR "nursing"[tiab] OR "nurse-led"[tiab])
	4	(Randomized Controlled Trial[pt] OR Controlled Clinical Trial[pt] OR randomized[tiab] OR placebo[tiab] OR trial[tiab] OR groups[tiab]) NOT (animals[mh] NOT humans[mh])
	5	#1 AND #2 AND #3 AND #4
Embase	1	('brain injury'/exp OR 'head injury'/exp OR 'traumatic brain injury*':ti,ab,kw OR 'severe TBI':ti,ab,kw OR 'head trauma':ti,ab,kw)
	2	('cognitive rehabilitation'/exp OR 'sensation'/exp OR 'arousal'/exp OR 'sensory stimulation':ti,ab,kw OR 'multisensory stimulation':ti,ab,kw OR 'cognitive stimulation':ti,ab,kw OR 'coma arousal':ti,ab,kw OR 'reality orientation':ti,ab,kw)
	3	('nurse'/exp OR 'nursing care'/exp OR 'nurse':ti,ab,kw OR 'nursing':ti,ab,kw OR 'nurse led':ti,ab,kw)

4	('randomized controlled trial'/exp OR 'controlled clinical trial'/exp OR random*:ti,ab,kw OR trial:ti,ab,kw OR placebo:ti,ab,kw)

CNKI: TKA=title OR keyword OR abstract, %= 'str' indicates that the relevant record matches str.

Web of Science: TS=title OR abstract OR keyword.

Embase: ti=title, ab=abstract, kw=keyword, exp=Emtree term (similar to Mesh term in Pubmed).

Cochrane Central Registry of Controlled Trials (CENTRAL): ti=title, ab=abstract, kw=keyword.

Scopus: TITLE-ABS-KEY=title OR abstract OR keyword.

PubMed: [mh]=MeSH Heading (Medical Subject Headings), [tiab]=title OR abstract, [pt]=publication type.
